# Structural basis of human Na_v_1.5 gating mechanisms

**DOI:** 10.21203/rs.3.rs-3985999/v1

**Published:** 2024-04-11

**Authors:** Rupam Biswas, Ana López-Serrano, Hsiang-Ling Huang, Angelina Ramirez-Navarro, Giovanna Grandinetti, Sarah Heissler, Isabelle Deschênes, Krishna Chinthalapudi

**Affiliations:** The Ohio State University; The Ohio State University; The Ohio State University; The Ohio State University; The Ohio State University; The Ohio State University; The Ohio State University; The Ohio State University

## Abstract

Voltage-gated Na_v_1.5 channels are central to the generation and propagation of cardiac action potentials^1^. Aberrations in their function are associated with a wide spectrum of cardiac diseases including arrhythmias and heart failure^2–5^. Despite decades of progress in Na_v_1.5 biology^6–8^, the lack of structural insights into intracellular regions has hampered our understanding of its gating mechanisms. Here we present three cryo-EM structures of human Na_v_1.5 in previously unanticipated open states, revealing sequential conformational changes in gating charges of the voltage-sensing domains (VSDs) and several intracellular regions. Despite the channel being in the open state, these structures show the IFM motif repositioned in the receptor site but not dislodged. In particular, our structural findings highlight a dynamic C-terminal domain (CTD) and III-IV linker interaction, which regulates the conformation of VSDs and pore opening. Electrophysiological studies confirm that disrupting this interaction results in the fast inactivation of Na_v_1.5. Together, our structure-function studies establish a foundation for understanding the gating mechanisms of Na_v_1.5 and the mechanisms underlying CTD-related channelopathies.

The cardiac voltage-gated sodium (Na_v_) channel Na_v_1.5 is essential for cardiac excitability and conduction^1^. Na_v_1.5 initiates the rapid influx of sodium ions from the extracellular region into the cytosol during the upstroke of the cardiac action potential. Alteration in Na^+^ currents has been extensively implicated in the etiology of arrhythmias^9,10^. Hundreds of mutations in *SCN5A*, the gene encoding for Na_v_1.5, have been linked to arrhythmia syndromes such as Brugada Syndrome (BrS) and Long QT Syndrome type 3 (LQT3) among others^11^. Hence, understanding the structure and function of Na_v_1.5 is pivotal for unraveling the molecular basis of cardiac electrical activity and developing targeted therapies for arrhythmias and other related disorders.

Eukaryotic Na_v_ channels are composed of a pore-forming α subunit and auxiliary β subunits^12^. The α subunit consists of four homologous domains (D_I_ to D_IV_) that share significant similarities yet exhibit different functions. Each domain is composed of six transmembrane segments referred to as S1 to S6. The voltage-sensing domain (VSD, S1 to S4) and the pore-forming domain (PD, S5 to S6) are arranged in a pseudo-tetrameric manner^13,14^. The N-terminal domain (NTD) and the C-terminal domain (CTD) are located in the cytosol. During a cardiac action potential, Na_v_ channels get activated and rapidly transition into a non-conductive state known as the fast inactivated state^7^. This transition occurs within 2–3 milliseconds following fast activation and it returns to the resting state upon repolarization^7^.

Functional studies identified the Isoleucine-Phenylalanine-Methionine (IFM) motif in the linker between D_III_ and D_IV_ (III-IV linker) as crucial for fast inactivation^15^. Published structures of Na_v_ channels showed that the IFM motif gets sequestered into a receptor binding pocket distant from the pore which leads to the proposed ‘door wedge’ model for fast inactivation^8,16–19^. It was also shown that mutations that hinder the interactions between the IFM motif and its receptor binding pocket allosterically propagate to the S6 segments^8,20^. Another model suggests that residues located at the intracellular end of the pore-forming S6 segment, rather than the IFM motif, cause fast inactivation^21^. Published work also implied a role for the CTD in regulating sodium channel function^2,22–25^. Indeed, the replacement of the native CTD of Na_v_1.5 with that of Na_v_1.4 confers inactivation kinetics that mimics Na_v_1.4 and vice versa^23,24,26^. Additionally, the CTD has been shown to be a hotspot for LQT3 mutations that affect fast inactivation^27^. Although electrophysiological and structural studies have identified critical functional elements, the molecular mechanisms underlying fast inactivation and the role of the CTD have yet to be established in the framework of full-length structures of Na_v_1.5, since these intracellular domains have not been resolved in published structures.

Here, we report three high-resolution cryo-electron microscopy (cryo-EM) structures of human full-length Na_v_1.5 (hNa_v_1.5) in the open state. Although there has been significant advancement in the understanding of Na_v_1.5 function over the past three decades, our insights into fast inactivation mechanisms remain limited due to incomplete structural knowledge of the intracellular regions. Our structures show previously unseen intracellular regions of Na_v_1.5 including the NTD and the CTD. The open state structures feature significant conformational changes in the VSDs and exhibit high plasticity in the positioning of the CTD and the III-IV linker. Site-directed mutagenesis coupled with electrophysiological measurements indicate that disrupting the interaction between the CTD and the III-IV linker can result in compromised fast inactivation mechanisms. In summary, our findings elucidate a mechanism for fast inactivation and the role of CTD in maintaining the open state conformation.

## The overall architecture of full-length hNa_v_1.5

**Figure 1:**
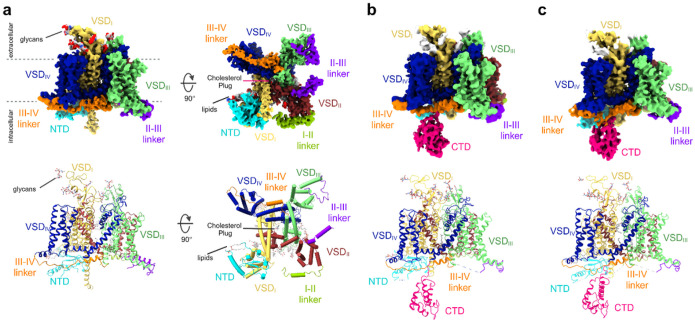
Cryo-EM structures of full-length hNa_v_1.5. **a**, Side (left) and intracellular (right) view of the cryo-EM reconstruction of Model-I. Individual domains and inter-domain linkers are segmented and color-coded. The lower panel depicts the atomic structure of Model-I including the resolved inter-domain linkers, lipid molecules, the cholesterol plug, and covalently attached glycans. The structural features are segmented and color-coded according to the density map. **b**, Side view of the cryo-EM reconstruction of Model-II. Color-coded according to (a). The CTD is segmented and shown in pink. The lower panel shows the atomic structure of Model II. **c**, Side view of the cryo-EM reconstruction (top) and atomic structure (bottom) of Model-III. Color-coded according to (b).

We purified recombinant full-length hNa_v_1.5 from Expi293 cells (Extended Data Fig. 1a). Negative stain electron microscopy and SDS-PAGE demonstrated the purity of our protein preparations (Extended Data Fig. 1b,c). We used optimal screened grids for cryo-EM data collection. Analysis of our cryo-EM data showed significant heterogeneity owing to the flexible intracellular regions of hNa_v_1.5. We assessed the structural heterogeneity by establishing a data processing workflow where particles were clustered into three classes (classes 1–3) based on the presence of intracellular features (Extended Data Fig. 2a). Class-1 contained high-resolution features of the core sodium channel. Class-2 and class-3 additionally contained the intracellular regions of hNa_v_1.5 (Extended Data Fig. 2a). The processed data yielded structures of hNa_v_1.5 with overall resolutions of ~3.2 Å for class-1 (Model-I), ~3.3 Å for class-2 (Model-II), and ~3.6 Å for class-3 (Model-III) (Extended Data Figs. 2b and 3a-d). Model-I comprises a total of 1353 residues and contains the transmembrane core, extracellular regions, the III-IV linker, portions of the I-II and II-III linker, and the NTD (Fig. 1a). Model-II and Model-III consist of 1418 residues each and contain the CTD in addition to all structural features observed in Model-I (Fig. 1b,c). The cryo-EM reconstructions show unambiguous densities for the VSDs, the selectivity filter, the pore-lining S6 segments, and the NTD (Extended Data Figs. 4–7). The CTD and other intracellular regions exhibit lower local resolutions due to their flexibility (Extended Data Figs. 2 and 7).

**Figure 2:**
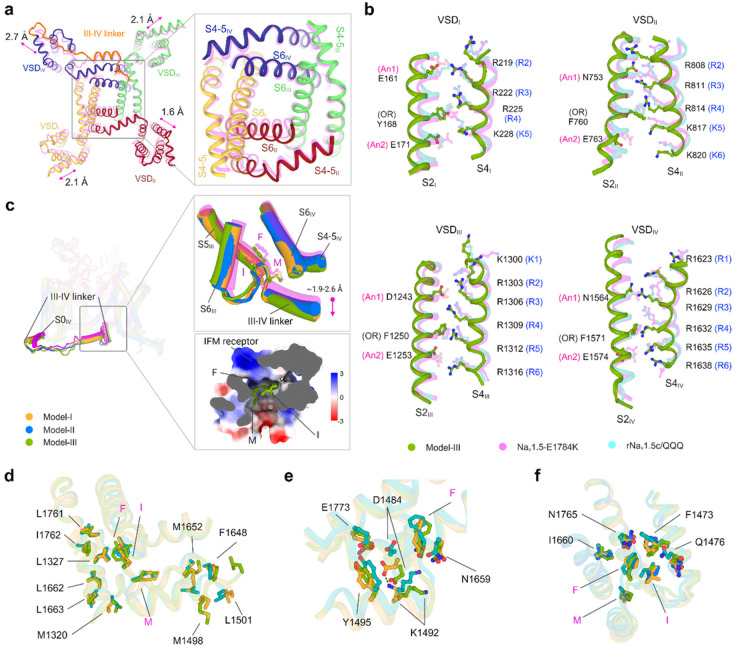
Insights into the pore domains, VSDs, and III-IV linkers of hNa_v_1.5. **a**, The intracellular view of the structural superimposition of Model-I (bold color) and Na_v_1.5-E1784K (PDB ID: 7DTC, transparent magenta) displays a lateral dilation of the VSDs. The inset shows the dilation of the PD. **b**, Comparative analysis of the conformation of GC residues of individual VSDs in Model-III, Na_v_1.5-E1784K, and rNa_v_1.5c/QQQ (PDB ID: 7FBS). GC residues are shown in stick representation. An1 and An2 denote anion1 and anion2, respectively. OR is occluding residue. For clarity, only the S2 and S4 segments of all the VSDs are shown. **c**, Superimposition of Model-I, Model-II, Model-III, and Na_v_1.5-E1784K (magenta). The III-IV linker and its connecting S0_IV_ helix are highlighted. The conformational changes of the IFM and III-IV linker helix are shown in the upper right inset. The lower inset displays the electrostatic surface potential of the IMF receptor bound to the IFM motif of Model-III. **d**, Hydrophobic interactions at the IMF receptor of Model-I (bright orange), Model-III (splitpea), and Na_v_1.5-E1784K (teal). **e**, Polar interactions at the IMF receptor. D1484 moves downward from NaV1.5-E1784K to Model-III. D1484 and K1492 form a salt bridge in Model-I. **f**, Interaction between the IFM motif and the receptor pocket residues. Key residues are shown in stick representation.

The a-subunit of the hNa_v_1.5 shows a pseudo-tetrameric structure consisting of the D_I_ to D_IV_ domains in a domain-swapped manner (Fig. 1a–c), consistent with the overall architecture observed in published structures of Na_v_ channels^6,8,16–18,28,29^. The NTD and the CTD emanate from the transmembrane portion of the channel on the intracellular side. The NTD is located at the base of VSD_I_. The CTD is a compact domain that is connected to the S6-helix of the D_IV_ via a flexible linker. The position of the CTD is variable with respect to the core transmembrane domain (Fig. 1b,c). Model-I exhibits a root-mean-square deviation (RMSD) of 0.6 Å to 0.8 Å over about 1050 Cα residues when compared to Model-II and Model-III. The RMSD between Model-II and Model-III is 0.7 Å for 1113 Cα residues. The transmembrane cores of Models-I to -III have an RMSD of 1.6 Å to 1.8 Å over nearly 1100 C-α residues compared to the human Na_v_1.5-E1784K structure (PDB ID: 7DTC) and show an outward movement of ~2 Å of the individual VSDs (Fig. 2a and Supplementary Video 1). This structural change is accompanied by the expansion of the pore domain (Fig. 2a and Supplementary Video 1).

## Sequential activation of gating charges is coupled with fast inactivation

Capturing VSDs in different confirmations is necessary to understand the precise activation and inactivation mechanisms of Na_v_1.5. The VSDs in the previous open and inactive state structures of Na_v_1.5 show similar conformations (Extended Data Fig. 8)^17,20,30^. The superposition of our models showed a gradual upward movement of gating charges (GCs) in VSD_II_ and VSD_IV_ caused by altered sidechain conformations (Extended Data Figs. 9 and 10). Specifically, multiple salt bridge interactions were rearranged, and two unique p-cation interactions were established between occluding residue (OR) and R4 of Model-III (Extended Data Fig. 9). Only three GCs in VSD_II_ and VSD_IV_ with minimal outward translocation are positioned above the OR, indicating a partially depolarized state. VSD_I_ and VSD_III_ are in the depolarized conformation with three and four GCs positioned above the OR, respectively. This suggests a hierarchical pattern (S4_III_ > S4_I_ > S4_II_ > S4_IV_) of VSD activation states as predicted in previous functional studies^31,32^.

Classification of our open state structures based on the conformational spectrum of the VSDs revealed that Model-III represents a late activated state, while Models II and I represent early inactivated states of hNa_v_1.5. Comparison of our structures with that of Na_v_1.5-E1784K (PDB ID: 7DTC; intermediate inactivated state) revealed a sequential shift in the side chains of GCs as they transition from the open to the fast inactivated state (Fig. 2a and Extended Data Figs. 10 and 11). This observation provides a structural framework for fast inactivation in the order of Model-III > Model-II > Model-I > Na_v_1.5-E1784K.

## The III-IV linker and interaction with CTD

**Figure 3:**
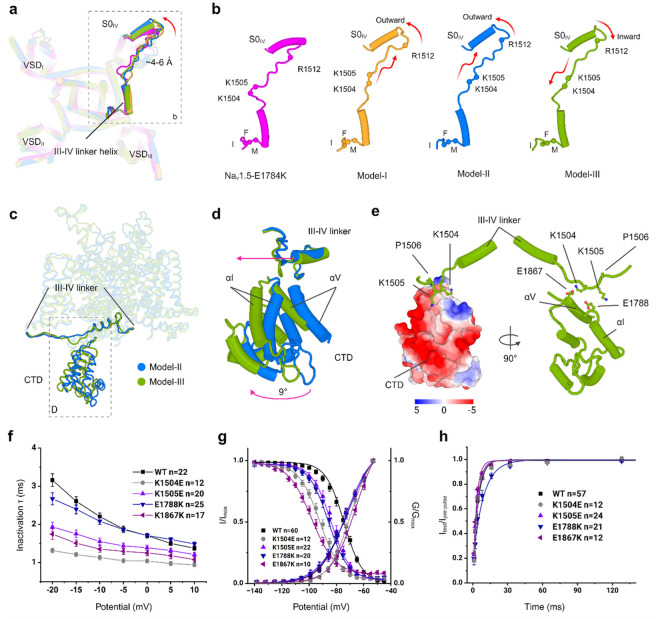
Molecular interactions near the IFM motif, as well as the conformational dynamics of III-IV linker and CTD. **a**, The III-IV linker and outward tilting of the S0_IV_ helix are highlighted in the overlay of Model-I, Model-II, Model-III, and Na_v_1.5-E1784K. **b**, The translation of the flexible loop of the III-IV linker is associated with the tilting of the S0_IV_ helix. The sphere represents the positions of three mutational hotspot residues. **c**, Positioning of the III-IV linker and CTD in Model-II and Model-III. **d**, The position of the CTD differs by > 9° between Model II and Model III. **e**, Key residues, K1504 and K1505, of the III-IV linker are in proximity to the negatively charged surface of CTD (left) and near to E1867 and E1788 residues of the CTD of Model-III (right). **f**, Electrophysiological recordings of current-voltage relationships displayed a faster time course of inactivation for K1504E, K1505E, and E1867K. **g**, Charge-reversal mutants K1504E, K1505E, E1788K, and E1867K cause a hyperpolarized shift in steady-state inactivation. A depolarized shift in the conductance curve was seen for K1504E and E1867K. **h**, E1867K displayed a slower recovery from inactivation.

The positioning of the CTD with respect to the III-IV linker and the transmembrane core is crucial for the working mechanisms of hNa_v_1.5. The IFM motif is loosely docked into the hydrophobic receptor pocket formed by the S4_III_-S5_III_ linker and the intracellular ends of S5_IV_ and S6_IV_ (Fig. 2c). This positioning differs from other mammalian Na_v_1.5 structures as follows. First, the short α-helix that immediately follows the IFM motif and the IFM receptor exhibits a downward displacement (Fig. 2c). The IFM motif is mostly engaged in hydrophobic interactions within the receptor binding pocket (Fig. 2d). The stability of the pocket is primarily maintained by a cluster of hydrophobic residues and polar contacts (Fig. 2d,e). Altered side chain conformations of N1765, F1473, and Q1476 cause a downward shift in the IFM motif without fully displacing it from the receptor (Fig. 2f). This structural rearrangement is further stabilized by the interaction between D1484 and K1492, located in the short α-helix (Fig. 2e). Second, we observed a significant change in the conformation of the S0_IV_ helix and the connecting loop of the III-IV linker. The N-terminal end of the S0_IV_ moved ~4 to 6 Å outward which caused a significant displacement of the flexible loop of the III-IV linker (Fig. 3a,b). A consequence of this transition is that the IFM motif is repositioned but not displaced from the receptor despite the channel being in the open state (Supplementary Video 1).

Notably, we have resolved 100 residues of the previously unseen CTD in Model-II and Model-III in two different conformations. The CTD is positioned closer to the III-IV linker in Model III and away from the III-IV linker in Model II. The superimposition of Model-II and Model-III shows that the position of the CTD differs by ~9° (Fig. 3c,d and Supplementary Video 2). To understand the direction and magnitude of CTD flexibility, we performed normal mode analysis (NMA) on Model-II and Model-III. This analysis revealed similar dynamics of the CTD in both structures (Supplementary Video 3). The comparison with the CTD in Na_v_PaS and Na_v_Pas-Na_v_1.7 chimera revealed significant differences in the positioning of the III-IV linker. In our structures, the CTD interacts with the flexible loop of the III-IV linker rather than being positioned on the short α-helix of the III-IV linker. This results in the bending of the flexible loop of the III-IV linker at residues K1504 and K1505 and causes an inward movement of the S0_IV_ helix and the short α-helix of the III-IV linker (Fig. 3b,e and Supplementary Video 2). In Model III, both residues are positioned proximate to the negatively charged surface residues, specifically E1788 on the αI helix and E1867 on the αV helix, within the CTD (Fig. 3b). Site-directed mutagenesis coupled with electrophysiological measurements were used to assess the importance of this interface in the inactivation kinetics of hNa_v_1.5. Our data revealed that charge reversal mutations K1540E and K1505E in the III-IV linker and E1788K and E1867K in the CTD altered hNa_v_1.5 inactivation parameters. Mutations K1504E, K1505E and E1867K resulted in a faster time course of inactivation compared to WT (Fig. 3f, Extended Data Fig. 12a and , and Extended Data Table 2). Additionally, all four mutants produced destabilization in inactivation as illustrated by a significant hyperpolarized shift in steady-state inactivation (Fig. 3g and Extended Data Table 2). We also assessed the recovery from inactivation and one of the mutants, E1788K, displayed a slower recovery (Fig. 3h and Extended Data Table 2). While none of the mutants significantly affected current densities (Extended Data Fig. 12b), K1504E and E1867K exhibited a depolarized shift in the conductance curve (Fig. 3g and Extended Data Table 2), suggesting delayed activation. Our results show that the dynamics between the CTD and the III-IV linker are crucial for the transition of hNa_v_1.5 from the open state to the inactivated state during the kinetic cycle.

## Conformation of the two-tier hydrophobic activation gate in the open state

**Figure 4:**
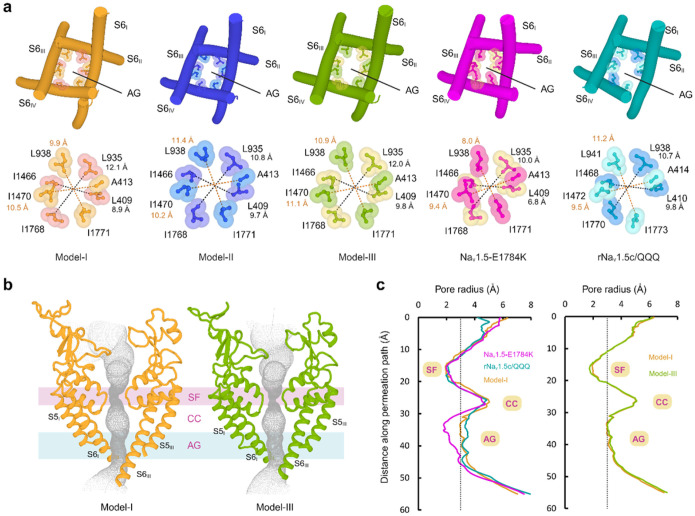
Activation gate diameter in open state. **a,** Comparison of the activation gate diameter in Model-I, Model-II, and Model-III, Na_v_1.5-E1784K (PDB: 7DTC), and rNa_v_1.5c/QQQ (PDB: 7FBS). The black and orange dashed lines represent the diameter at the top and bottom layers of the activation gate, respectively. **b**, The permeation paths of Model-I and Model-III are shown as grey dots. SF: selectivity filter, CC: central cavity, AG: activation gate. **c**, The corresponding pore radii are compared with that of Na_v_1.5-E1784K and rNa_v_1.5c/QQQ.

The opening and closing of the activation gate regulate the influx of sodium ions into the cells. In our structures, the activation gate is open. We used the position of two hydrophobic rings at the lower part of the S6 helices as a reference for the comparison of the activation gate in our three structures with the Na_v_1.5-E1784K (PDB ID: 7DTC, intermediate inactive state) and rNa_v_1.5c/QQQ (PDB ID: 7FBS, open state) structures^21^. The residues on the top layer are L409, L935, I1466, and I1768. The bottom layer residues are A413, L938, I1470, and I1771. In the open state structure of rNa_v_1.5c/QQQ, the average pore diameter is 10.2 Å at the top layer and 10.3 Å at the bottom layer (Fig. 4, A and C). The average pore diameter in the upper and lower layer of Na_v_1.5-E1784K is 8.4 Å and 8.7 Å (Fig. 4a,c) indicative of an intermediate state. Comparison with Model-I showed that our average pore diameters at the top and bottom layer are 10.5 Å and 10.2 Å and closely resemble those of rNa_v_1.5c/QQQ (Fig. 4a–c)^20^. In Model-II, the diameter of the top layer decreased to 10.2 Å and the diameter of the bottom layer increased to 10.8 Å in Model-II (Fig. 4a and Extended Data Fig. 13). We observed an even larger activation gate diameter in Model-III, measuring 10.9 A at the top layer and 11.0 Å at the bottom layer (Fig. 4a–c). Thus, we propose that Models I to III represent open state conformation of hNa_V_1.5. Notably, in all our structures, we observed a synchronized movement of the S6 helices, leading to an equivalent pore diameter of the activation gate (Supplementary Video 1). A van der Waals space-filling model shows that hydrated Na^+^ fits through the orifice of the activation gate in our open state structures (Extended Data Fig. 14). In summary, our structures show activation gates with average pore diameters that exceed those of published Na_v_1.5 structures. We attribute this characteristic to the presence of untruncated, native intracellular regions.

## Distributions of disease-causing mutations in the NTD and CTD

**Figure 5:**
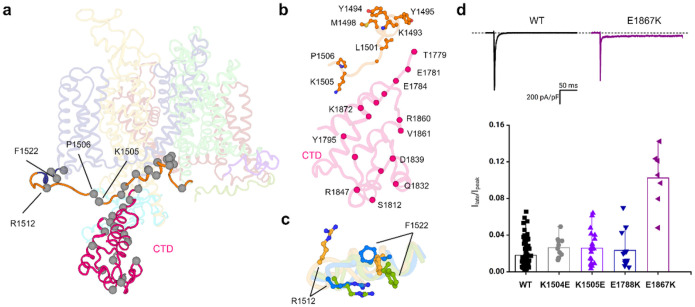
Structural mapping of mutations linked to BrS and LQT3. **a**, Structural mapping of mutation hotspot residues (sphere). **b**, A cluster of selected mutations associated with BrS and LQT3 in the region of III-IV linker and CTD. **c**, Interaction between R1512 and F1522 in Model-I (bright orange), Model-II (marine), and Model-III (split pea). The π-cation interaction occurs exclusively in Model-III. **d**,E1867K mutation presented a significant increase in persistent current, but no change in persistent current was observed in K1504E, K1505E, and E1788K.

Previous Na_v_1.5 structures have not resolved intracellular regions that contain the majority of Na_v_1.5 disease-causing mutations. However, our cryo-EM structures include the previously unresolved NTD and the CTD of hNa_v_1.5. This allowed us to systematically map the disease-causing mutations linked to LQT3 and BrS in these regions. Analysis of mutations in the NTD and its interacting S6_I_ helix, as well as in the CTD and its interacting III-IV linker, showed a distinct pattern of distribution in the structures (Fig. 5a and Extended Data Fig. 15). We found that mutations associated with LQT3 and BrS are dispersed evenly in the NTD (Extended Data Fig. 15) while a cluster of mutations is present in the III-IV linker and the adjacent region of the CTD (Fig. 5a,b).

Mutations in the bending region of the III-IV linker, specifically at K1505 and P1506 of the KPQ sequence, can lead to LQT3 and BrS (Figs. 3b and 5b)^3–5,33,34^. P1506 is crucial in shaping the curvature of the III-IV linker, which enables the interaction between K1504-K1505 and the CTD (Supplementary Video 2). Our structures further revealed an important mutational hotspot at the C-terminal end of the III-IV linker and its connecting S0_IV_ helix where residue R1512 engages in a p-cation interaction with F1522 in Model-III (Fig. 5c and Supplementary Videos 1 and 2). This interaction is disrupted in both Model-II and Model-I, suggesting that the p-cation interaction is transient and reliant on the conformation of the S0_IV_ helix. Mutation R1512W can lead to a robust p-stacking interaction with F1522 that limits the flexibility of the S0_IV_ helix and its connecting III-IV linker and leads to the reported changes in hNa_v_1.5 kinetics in BrS^35,36^. It is well-established that mutations leading to LQT3, found in regions with inactivation, often present with an increase in persistent current. Here, we observed that the introduction of mutant E1867K resulted in a significant increase in late current (Fig. 5d), further supporting the contribution of this region to cardiac channelopathies.

## Conclusion

**Figure 6:**
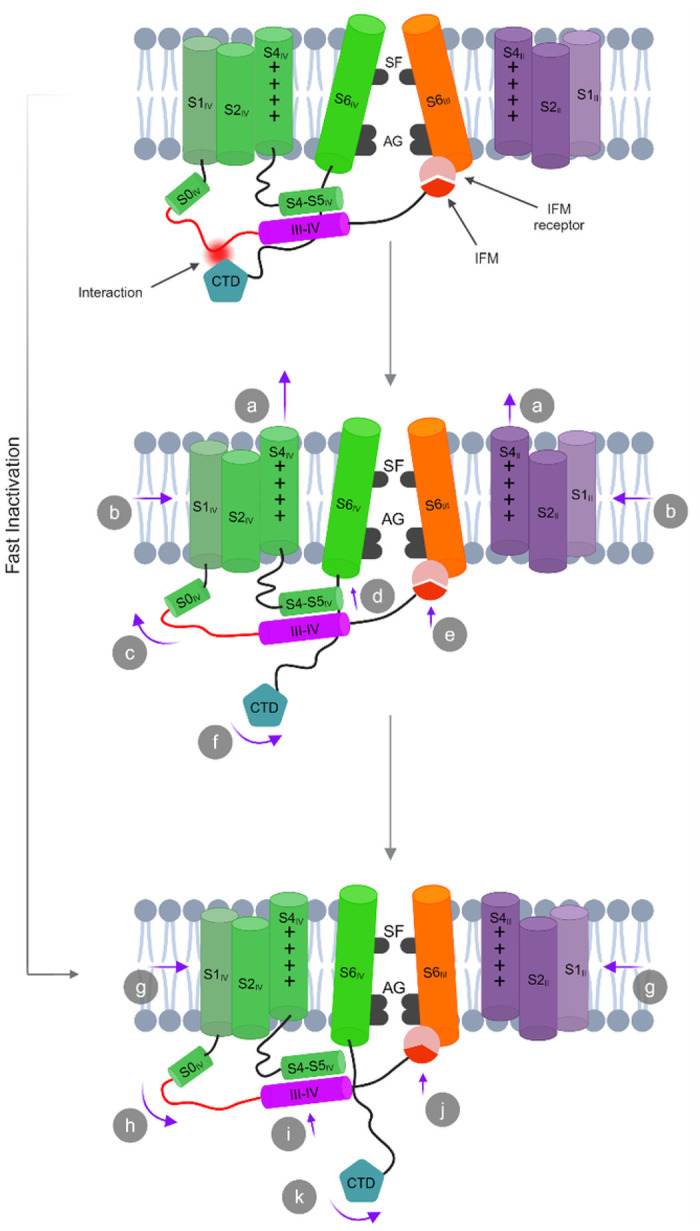
Mechanism of fast inactivation. Transition from the open state to the inactivated state. The conformational changes in each state are assigned with letters. (a) S2_II_ and S4_IV_ sequentially move upward, transitioning from a partially depolarized to a fully depolarized conformation. (b) Overall dilation is reduced. (c) Then the S0_IV_ helix is slanted outward and forms an extended conformation of the flexible loop of the III-IV linker. (d) S4-S5_IV_ linker and III-IV linker helices move upward. (e) IFM motif undergoes a transition from a loosely bound state to a semi-tight conformation. (f) The CTD partially moves away from the III-IV linker, resulting in a loss of electrostatic interactions. Transition from the intermediate state to inactivated state: (g) A further reduction in overall dilation. (h) S0_IV_ is slanted inward and forms a relaxed conformation of the flexible loop of the III-IV linker. (i) S4-S5_IV_ linker and III-IV linker helices move further upward. (j) IFM motif undergoes a shift from a semi-tight conformation to a tightly bound state. (k) CTD moves further away and retains a dynamic conformation. Schematics are not drawn to scale.

Our cryo-EM studies resolved novel structural features of hNa_v_1.5, highlighting conformational dynamics around the VSDs and CTD. Our native, untruncated structures of hNa_v_1.5 are captured in the open state. Importantly, the IFM motif in our structures is repositioned within its receptor but not displaced, providing crucial insights into the fast inactivation mechanism. The consequence of this repositioning of the IFM and the III-IV linker are transduced allosterically to the S6 segments thereby facilitating the opening of the activation gate. These allosteric conformational changes may also be attributed to the overall increased lateral dilation of the structures. Together, we demonstrated how the expansion of the activation gate and pore domain are interconnected, along with the overall expansion of the entire structure through concerted movements of the pore-forming segments. Additionally, in the open state structure of hNa_v_1.5, we identified a partially activated GC conformation in VSD_II_ and VSD_IV_ that is coupled with the interaction between the III-IV linker and the CTD. We suggest that this interaction stabilizes the open state of the activation gate. Disruption of this interaction leads to the accelerated closing of the activation gate, underscoring the essential role of CTD in fast inactivation regulation (Fig. 6).

Our structures capture the unanticipated kinetic states of the hNa_v_1.5, showing the conformational changes from the open state to the inactivated state. Notably, these structures further enabled us to pinpoint several key residues related to LQT3 and BrS in the NTD, the III-IV linker, and the CTD. Overall, our study sheds light on the electromechanical coupling among VSDs, the III-IV linker, and the S5-S6 segments, and their previously unresolved relationship with the CTD. In conclusion, our findings reveal the potential mechanism of fast inactivation and provide an essential roadmap for studying inactivation mechanisms and regulation of the channel by its numerous protein partners which interact predominantly with the intracellular regions of Na_v_1.5.

## Methods

### Cloning of the full-length human Na_v_1.5 expression vector

Codon-optimized full-length human Na_v_1.5 (hNa_v_1.5) cDNA was synthesized (GenScript, NJ, USA) and cloned into a modified pCDNA3.1 vector containing a twin-Strep tag and a TEV site at the N-terminus and an HRV 3C site and a FLAG tag at the C-terminus of the channel cDNA. Mutations K1504E, K1505E, E1788K, and E1867K were introduced in the Na_v_1.5 cDNA via site-directed mutagenesis (Quick change II mutagenesis kit, Agilent, CA, USA). All vectors were verified by Sanger sequencing.

### Expression of hNa_v_1.5

Full-length human Na_v_1.5 was recombinantly produced in Expi293F GnTI- suspension cells (ThermoFisher). Cells were grown in Expi293 expression medium (ThermoFisher) at 37°C in 8% CO2. Transient transfections of pCDNA3.1-hNa_v_1.5 were done with ExpiFectmaine 293 (ThermoFisher). Cells were harvested ~ 48 h post-transfection and the cell pellets were flash-frozen in liquid nitrogen.

### Purification of the full-length human cardiac sodium channel

Pellets from 6 litres of cells were used for the purification of hNa_v_1.5. The cell pellet was first washed with 1X PBS and subsequently suspended in lysis buffer containing 25 mM HEPES (pH 7.4), 150 mM NaCl, 1 mM EGTA, and 10% glycerol (buffer A). A protease inhibitor cocktail was added to the suspension before homogenization. The membrane fraction was collected through ultracentrifugation, then resuspended and dissolved in buffer A supplemented with protease inhibitors, 1% (w/v) n-dodecyl-β-D maltopyranoside (DDM, Anatrace), and 0.1% (w/v) cholesteryl hemisuccinate (CHS, Anatrace). The suspension was gently stirred at 4°C for 2 hours. The cell lysate was subjected to ultracentrifugation (30,000 RPM, 4° C, 30 min). The supernatant was incubated for 2 hours with 5 mL of anti-Flag M2 affinity gel that had been equilibrated in advance with buffer B (buffer A supplemented with 0.06% (w/v) glycol-diosgenin (GDN, Anatrace) and protease inhibitor cocktail). The protein-bound FLAG M2 affinity gel was washed with 10-column volumes (CV) of buffer B. The bound protein was eluted using buffer B supplemented with 200 μg/ml of FLAG peptide. The eluent was incubated for 1.5 hours with 3 mL of Strep-Tactin XT 4Flow (IBA) high-capacity resin that had been pre-equilibrated with buffer B. The resin was rinsed with 5 CVs of buffer B, followed by elution of the protein using buffer B containing 50 mM biotin (IBA). The eluted protein was concentrated using a 100-kDa cut-off concentrator (Millipore) and subsequently purified using a Superose 6 increase 10/300 gl column (Cytiva) in buffer C (25 mM HEPES at pH 7.4, 150 mM NaCl, 0.1 mM EGTA and 0.06% GDN). The peak fractions of the purified protein were pooled and concentrated to approximately 8 μM for cryo-EM analysis.

### Cell culture and expression of hNa_v_1.5

HEK293 cells were grown in DMEM (Gibco) high glucose supplemented with 10% fetal bovine serum (Gibco) and 1% penicillin-streptomycin (Sigma) at 37°C in 5% CO2. The optimized Nav1.5-WT and mutations were stably transfected into HEK293 cells. The transient transfection was performed by electroporation method for maximal transfection efficiency with ATx from MaxCyte (Gaithersburg, MD) according to the manufacturer’s instructions. Briefly, cells were dissociated at about 70% confluency and mixed with the target plasmid and transfection buffer. The concentration of the plasmid transfected was 200ng/ml in the electroporation buffer. Then, the cells were electroporated, followed by a 20 min recovery at 37°C and 5% CO2. Cells were then transferred to the maintenance medium for about 48 hours until electrophysiological recordings.

### Whole-cell electrophysiology

High-throughput automated patch-clamp experiments were performed using the SyncroPatch 384i (Nanion, Munich, Germany) as previously described^37^. Briefly, single-hole, low resistance recording chips from the same manufacturer were used to record sodium currents. Patch-clamp extracellular solution contained: 140mM NaCl, 4mM KCl, 2mM CaCl_2_, 1mM MgCl2, 5mM D-glucose, 10mM HEPES. The intracellular solution contained: 10mM HEPES, 10mM EGTA, 110mM CsF, 10mM NaCl, and 10mM CsCl. Protocol generation, data collection, and data analysis were performed on PatchController384 V.1.3.2 and DataController384 V1.10.1 (Nanion, Munich, Germany).

The current-voltage relationships of the sodium currents were recorded by holding the cells to −120mV and stepping from −80mV to +60mV in 5mV intervals (each step held for 30ms). For activation, the G/V curve was determined by fitting the linear part before the peak of the current-voltage curve with a Boltzmann function:

$$G(V)=G_{max}/\left(1+e^{-\left(V-V_{\text{half}}\right)/k}\right)$$

The time course of inactivation was obtained by fitting the current traces obtained from the current-voltage relationship with a single exponential fitting of each current trace from peak current to the end of the pulse (30ms) using the following equation:

 Iinact = A1 e (−t/τ)

where A is the amplitude, τ is the time constant, I is the current, and t is the time.

The recovery from inactivation was recorded with a two-pulse protocol. The pre-pulse and the test-pulse duration are 30ms, stepping from −120mV to −30mV. The interval between the two pulses ranges from 1ms to 250ms. Currents from the recovery from inactivation were fitted to the following equation:

$$I_{\text{test}}/I_{pre-\text{pulse}}=1-{\text{e}}^{-\text{t}/\tau_{rec}}$$

The steady-state inactivation was studied with a 500 ms pre-pulse ranging from −140mV to −30mV, followed by a 30ms test pulse stepping from −120mV to −30mV. The currents for the steady-state inactivation were fit to a Boltzmann distribution using the following equation:

I/Imax=(1+e(V−V12)/kv)−1

The late Na current was recorded with a 300ms pulse ranging from −120mV to −30mV. Late current was calculated from the percentage of the current measured at 250ms to the peak current.

The fitting curves for steady-state inactivation and recovery from inactivation analysis were generated with Origin 10.1.1 software (OriginLab Corporation, Northampton, MA). The leak subtraction protocol was used.

### Statistical analysis

Statistical analyses for electrophysiology data were performed using the standard statistical package in Origin 10.1.1 (OriginLab Corporation, Northampton, MA). The student’s t-test was performed at a significance level of p < 0.05 for a single comparison after a normality test with the Shapiro-Wilk method for sample sizes 7–50. Two-sided p-values less than 0.05 were considered statistically significant. Multiple comparisons with the different mutants were performed using one-way ANOVA, and p-values less than 0.05 were considered statistically significant. Results were presented as mean ± SEM.

### Sample preparation and cryo-EM data collection

Aliquots of 3.5 μL freshly purified hNa_v_1.5 was applied onto glow-discharged UltrAuFoil 300 mesh, R1.2/1.3 grids (Quantifoil). Grids were blotted for 3.5 s and plunge frozen in liquid ethane using Vitrobot Mark IV (Thermo Fisher). To evaluate the stability of the samples and the distribution of particles, the grids were screened using Glacios (Thermo Fisher Scientific) at 200 kV and equipped with a Falcon 3EC direct electron detector. Optimal grids based on the ice thickness and sample distribution were transferred to a Titan Krios G3i microscope (Thermo Fisher Scientific) operated at 300 kV and equipped with a K3 direct electron detector, a BioQuantum energy filter, and a Cs image corrector. A total of 4,967 movies were captured in super-resolution mode (pixel size of 0.4495 Å) at a magnification of 81,000x. The defocus range during collection varied from −0.5 to −2.5 μm. Each movie was exposed to a total electron dose of 60 e-/Å ^2^. The data collection was performed using EPU software (Thermo Fisher Scientific). The summed and dose-weighted micrographs were binned to a pixel size of 0.899 Å/pixel for further data processing. Data collection statistics are reported in Extended Data Table 1.

### Image processing and 3D reconstruction

All raw movies were aligned, drift-corrected, and dose-weighed using Patch Motion Correction in cryoSPARC v4.4.1^38^. Defocus and contrast transfer function (CTF) parameters were estimated using Patch CTF in cryoSPARC. The blob-picking module in cryoSPARC was used to generate templates for particle picking. A small set of particles for reference-free two-dimensional (2D) classifications was selected and subsequently used as templates for particle picking. After two rounds of iterative 2D classifications, selected particles were used for training in Topaz. The extracted particles were subjected to another two rounds of iterative 2D classifications. Sixteen well-aligned 2D classes were used as templates for ab initio 3D reconstruction with 281,571 particles in cryoSPARC. After one round of homogeneous refinement and non-uniform refinement, a 3D classification was performed to split the particles into four classes. Class-I with 100,809 particles showed clear features for the transmembrane region and extracellular domains. It was selected for non-uniform refinement and after optimizing and refining the defocus of particles, local refinement was performed by applying a soft mask to mask out the noisy regions and achieved a global resolution of 3.2 Å at a Fourier shell correlation (FSC) at 0.143. Class-II (75,907 particles) and Class-III (65,347 particles) showed extra density for the intracellular domains. Both classes were processed similarly to Class-1 with the exception that a soft binary mask which included the intracellular density was applied during the local refinement. A global resolution of 3.5 Å (Class-II) and 3.9 Å (Class-III) at a Fourier shell correlation (FSC) at 0.143 was achieved in cryoSPARC. Additionally, density modifications of the Class-II and Class-III maps were performed with the Resolve density modification tool in Phenix^39^. The final resolution after density modification was improved to 3.3 Å for Class-II and 3.6 Å for Class-III based on the gold-standard FSC=0.143 criteria. All the 3D maps were sharpened with the Autosharpen tool in Phenix and/or DeepEMhancer^40,41^. The 3DFSC server was used to calculate the 3D Fourier shell correlation and sphericity of each map^42^. The local resolution of the map was calculated using MonoRes in cryoSPARC. Data collection statistics and image processing summary are shown in Extended Data Table 1 and Extended Data Figs. 2 and 3.

### Model building, refinement, and validation

Three models of hNa_v_1.5 were built utilizing the sharpened maps and the AlphaFold2 model of hNa_v_1.5 as an initial template^43^. The full-length model was initially docked into the Class-I map using the Phenix Dock in Map tool. Subsequently, the unfitted regions of the docked model were removed in Coot. Iterative model building was performed using real-space refinement in Phenix and Coot to remove the outliers and improve the model refinement statistics. The model refined into the Class-I map has a total of 1353 residues. Additionally, 15 NAG and 11 lipid molecules were modeled into densities. The C-terminal sequences beyond A1778 were left unmodeled. For the maps corresponding to Classes-II and III, an additional 104 residues corresponding to the C-terminal domain (CTD) were built by using PDB ID: 4OVN as a template for rigid-body refinement of the backbone. It is important to note that the positioning of the side chain orientations are ambiguous at lower resolutions in the CTD. Thus, CTD residues were fitted and rotamers minimized using Namdinator^44^ and further refined in Phenix using real-space refinement with secondary structure and geometry restraints. Figures were prepared with the PyMOL (Schrödinger, LLC) and ChimeraX^45^. Final figures were assembled in BioRender (www.BioRender.com).

## Data Availability

The cryo-EM structures are deposited in the Protein Data Bank (PDB) and Electron Microscopy Data Bank (EMDB) under the following accession numbers: Model-I: PDB ID:8VYI, EMDB ID: EMD-43661; Model-II: PDB ID: 8VYJ, EMDB ID: EMD-43662; Model-III: PDB ID: 8VYK, EMDB ID: EMD-43663. Data supporting the findings of this study are available in the article and its Extended data and Supplementary information.
